# FAVA: high-quality functional association networks inferred from scRNA-seq and proteomics data

**DOI:** 10.1093/bioinformatics/btae010

**Published:** 2024-01-08

**Authors:** Mikaela Koutrouli, Katerina Nastou, Pau Piera Líndez, Robbin Bouwmeester, Simon Rasmussen, Lennart Martens, Lars Juhl Jensen

**Affiliations:** Novo Nordisk Foundation Center for Protein Research, Faculty of Health and Medical Sciences, University of Copenhagen, 2200 Copenhagen N, Denmark; Novo Nordisk Foundation Center for Protein Research, Faculty of Health and Medical Sciences, University of Copenhagen, 2200 Copenhagen N, Denmark; Novo Nordisk Foundation Center for Protein Research, Faculty of Health and Medical Sciences, University of Copenhagen, 2200 Copenhagen N, Denmark; VIB-UGent Center for Medical Biotechnology, VIB, 9052 Ghent, Belgium; Department of Biomolecular Medicine, Ghent University, 9052 Ghent, Belgium; Novo Nordisk Foundation Center for Protein Research, Faculty of Health and Medical Sciences, University of Copenhagen, 2200 Copenhagen N, Denmark; VIB-UGent Center for Medical Biotechnology, VIB, 9052 Ghent, Belgium; Department of Biomolecular Medicine, Ghent University, 9052 Ghent, Belgium; Novo Nordisk Foundation Center for Protein Research, Faculty of Health and Medical Sciences, University of Copenhagen, 2200 Copenhagen N, Denmark

## Abstract

**Motivation:**

Protein networks are commonly used for understanding how proteins interact. However, they are typically biased by data availability, favoring well-studied proteins with more interactions. To uncover functions of understudied proteins, we must use data that are not affected by this literature bias, such as single-cell RNA-seq and proteomics. Due to data sparseness and redundancy, functional association analysis becomes complex.

**Results:**

To address this, we have developed FAVA (Functional Associations using Variational Autoencoders), which compresses high-dimensional data into a low-dimensional space. FAVA infers networks from high-dimensional omics data with much higher accuracy than existing methods, across a diverse collection of real as well as simulated datasets. FAVA can process large datasets with over 0.5 million conditions and has predicted 4210 interactions between 1039 understudied proteins. Our findings showcase FAVA's capability to offer novel perspectives on protein interactions. FAVA functions within the scverse ecosystem, employing AnnData as its input source.

**Availability and implementation:**

Source code, documentation, and tutorials for FAVA are accessible on GitHub at https://github.com/mikelkou/fava. FAVA can also be installed and used via pip/PyPI <pip install favapy> as well as via the scverse ecosystem https://github.com/scverse/ecosystem-packages/tree/main/packages/favapy.

## 1 Introduction

Networks of physical and functional interactions among genes/proteins are widely used to understand the inner workings of cells and to visualize results from omics data, e.g. as obtained from transcriptomics and proteomics experiments. Unfortunately, most research is focused on the same 10% of human protein-coding genes (Lin *et al.* 2017, Stoeger *et al.* 2018, Oprea 2019). Thus, networks derived from the biomedical literature—whether through manual annotation or through automatic text mining—are heavily biased by this skewed availability of data. Networks obtained from databases such as STRING (Szklarczyk *et al.* 2021) consequently have many interactions for well-studied proteins. However, there are only very few interactions for understudied proteins—as defined by the “Illuminating the Druggable Genome” consortium—which are arguably the most interesting targets for network-based function prediction (Lin *et al.* 2017, Kustatscher *et al.* 2022a).

Recent publications (Kustatscher *et al.* 2022a, 2022b) as well as initiatives like the HUPO challenge grand project with the ambitious goal of “a function for every protein” (HPP Grand Project), highlight that the scientific community is increasingly prioritizing the identification of new information about understudied proteins. These proteins have been largely ignored in the past due to the Matthew effect in which proteins with proven disease importance are the subject of most studies (Kustatscher *et al.* 2022a). To create networks that also provide interactions for understudied proteins, one must focus on systematic high-throughput data, as these are inherently unaffected by literature bias.

One approach to address this is to use experimental approaches to systematically capture new information on e.g. physical protein interactions of understudied proteins (Luck *et al.* 2020). The hu.MAP 2.0 resource (Drew *et al.* 2021) is an example of this, providing protein complexes derived from over 15 000 proteomic experiments. By capturing physical interactions between proteins in a systematic manner, without literature bias, it provides protein interaction information for many completely uncharacterized proteins.

Complementary to this, technological advances such as RNA and mass spectrometry-based proteomics have enabled the discovery of protein functions and protein–protein relationships with greater accuracy on a large scale. Functional association network analysis is one popular approach for this; it works by linking genes that show similar expression patterns across many samples (van Dam *et al.* 2015, van Dam *et al.* 2017). Many computational methods have been developed for functional association network construction from bulk RNA-seq data (Serin *et al.* 2016), such as WGCNA (Langfelder and Horvath 2008). However, other types of data with higher resolution, such as single-cell RNA-seq (scRNA-seq) data, may allow better functional association networks to be constructed. ScRNA-seq provides unbiased data on gene expression at the level of individual cells, thus capturing differences between both cell types and cell states. However, methods optimized for bulk RNA-seq may not be successful in data produced by this technology due to its sparse and redundant nature.

As high-dimensional, sparse omics data are becoming increasingly common, in part due to single-cell technologies, new methods designed to handle such data are needed. Pearson correlation coefficient (PCC) is an often-utilized technique to establish functional association networks. Recently, more sophisticated computational methods have been developed for functional association network construction specifically from scRNA-seq data. Some of the state-of-the-art methods are the hdWGCNA (Morabito *et al.* 2022) and the scLink (Li and Li 2021). hdWGCNA makes use of the WGCNA (Langfelder and Horvath 2008) protocol to construct gene networks and to identify modules with specific provisions for high-dimensionality datasets. To enable this process in scRNA-seq data, hdWGCNA identifies similar cells, which are pooled, thereby dealing with sparsity. scLink (Li and Li 2021) manages sparsity by not considering all samples for every pair of genes, but instead focusing on the cells in which both genes are accurately measured. Additionally, the authors recommend creating functional association networks with scLink using only the top 500 most highly expressed genes.

While single-cell RNA expression data provide great detail, the correlation between RNA and protein levels is far from perfect (Edfors *et al.* 2016). It thus makes sense to consider also using proteomics data for creating functional association networks, although proteomics data at single-cell resolution currently remain a rarity [here we cite some of the most popular datasets (Cong *et al.* 2020, Schoof *et al.* 2021, Brunner *et al.* 2022, Huffman *et al.* 2023, Leduc *et al.* 2023, Montalvo *et al.* 2023)] with small numbers of cells (Petrosius and Schoof 2023). ScRNA-seq and proteomics can thus be viewed as two complementary types of data that can be used as starting points for predicting functional interactions, also for understudied proteins. Unfortunately, there are no computational methods designed to handle both types of data, taking advantage of their complementary nature. The biggest challenge remains that both scRNA-seq and proteomics datasets are very sparse (i.e. many transcripts/proteins are not observed in each cell/sample) and have high redundancy (i.e. many similar samples/cells are analyzed), both of which present problems for most analysis methods.

Dimensionality reduction can help address both sparsity and redundancy in data. By compressing the information into a lower-dimensional space, sparsity is eliminated by combining data from across multiple cells/samples. Redundancy is also inherently reduced, because data compression is specifically achieved by not storing the same information multiple times (van der Maaten *et al.* 2009). One can thus expect that applying dimensionality reduction to scRNA-seq and proteomics data provides a latent representation that is better suited for functional association prediction of interactions than the original data matrices.

**Figure 1.**
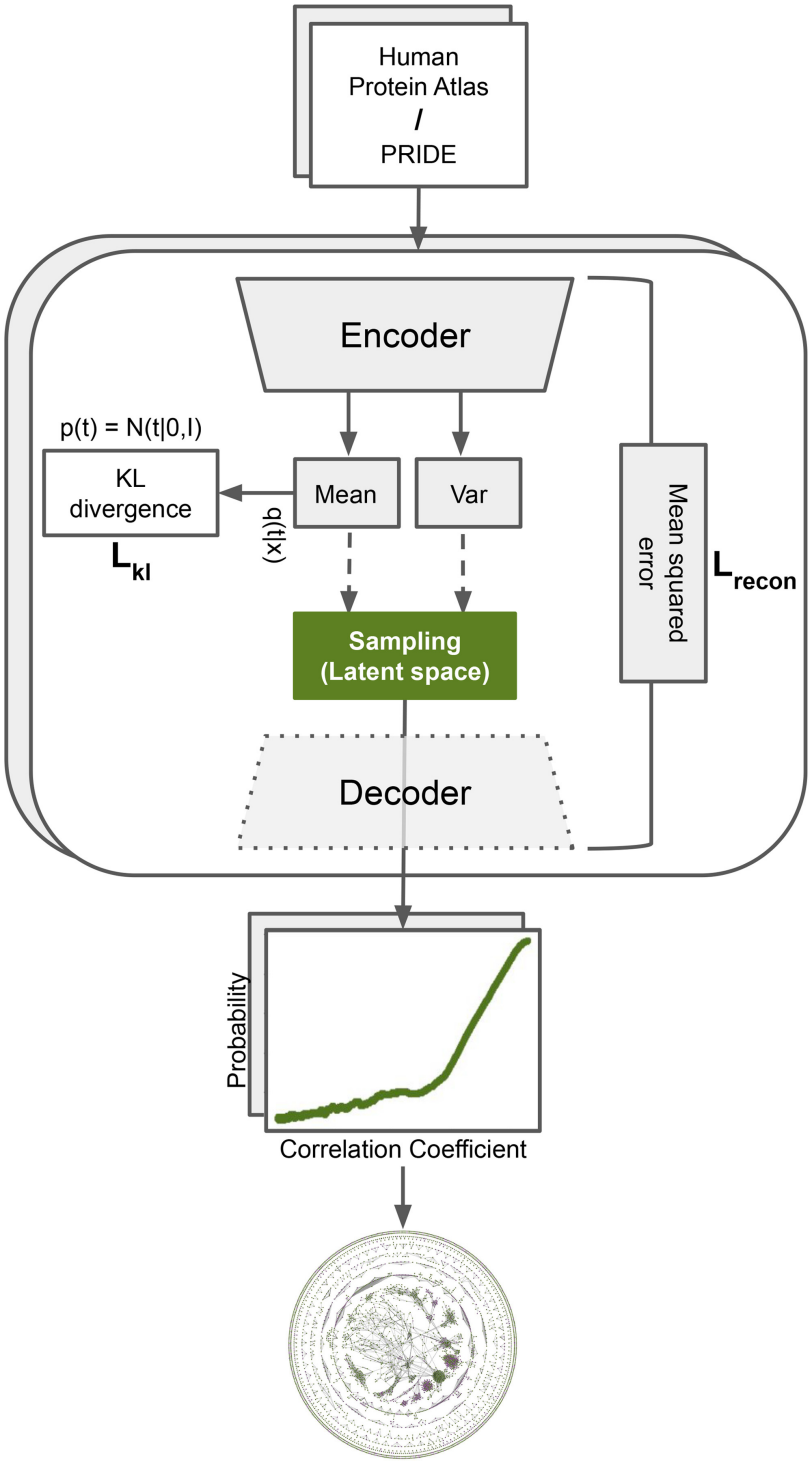
Overview of the FAVA method: from expression matrices to a combined network. The first step is to pre-process each input matrix before it is fed to a variational autoencoder (VAE). After pre-processing, the input matrix is used to train the VAE model, from which we obtain the latent space from the bottleneck layer. In this latent space, we calculate the Pearson correlation coefficient (PCC) for each pair of proteins, resulting in a functional association network. Next, we fit a calibration function to convert the PCC scores to posterior probabilities based on KEGG pathways. This pipeline is applied separately to each of the two matrices obtained from the Human Protein Atlas and PRIDE. Finally, we combine the posterior probabilities from the two networks to produce a single, combined network.

Dozens of dimensionality-reduction algorithms exist, which can be used to produce this latent space. These span linear methods such as truncated singular value decomposition (Klie *et al.* 2008, Franceschini *et al.* 2016), nonlinear transformations such as Uniform Manifold Approximation and Projection (UMAP) (McInnes *et al.* 2020), and deep generative models such as variational autoencoders (VAEs) (Kingma and Welling 2019). VAEs use two deep neural networks—an encoder and a decoder (as shown in Fig. 1)—to learn a representation from complex data without supervision (Kingma and Welling 2014). The encoder learns the input data and projects it into a normally distributed latent representation, parameterized by the mean (μ) and var (σ) layers, while the decoder attempts to reconstruct the input data from the instances sampled from the latent representation distributions. The encoder–decoder networks are simultaneously optimized to reconstruct the input data and regularize the latent representations. This ensures that the constructed latent representations follow Gaussian distributions, making them easier to interpret than those of other autoencoders, such as the ones described in (Hinton and Salakhutdinov 2006). Unlike e.g. singular value decomposition, VAEs can capture both linear and nonlinear relationships between the cells/samples in scRNA-seq and proteomics data. As a result, VAEs have become popular in the field of expression data (e.g. for normalization and visualization of scRNA-seq).

Here, we present FAVA, a method to infer Functional Associations using VAEs from omics data. We show that (i) the method can efficiently handle large-scale scRNA-seq and bulk proteomics data; (ii) it outperforms traditional functional association analysis and the state-of-the-art methods by a considerable margin on both real and simulated data; (iii) it can predict high-confidence functional associations; and (iv) the resulting networks provide good coverage also for understudied proteins with 4210 interactions between 1039 understudied proteins. FAVA is available as a Python package on PyPI, via the command pip install favapy, and it can be used through direct command line or as a module in Python scripts. In addition, the Python module can take as input either a counts/abundance matrix or an AnnData object (Virshup *et al.* 2021), making it compatible with the most popular single-cell analysis pipelines in Python [e.g. SCANPY (Wolf *et al.* 2018)].

## 2 Materials and methods

FAVA is a novel method for constructing functional association networks from complex omics datasets. Leveraging VAEs for dimensionality reduction, we introduce a robust approach to identify functional associations by analyzing single-cell RNA sequencing and bulk proteomics data, creating a combined network that highlights understudied proteins. Our method, distributed through the ‘favapy’ Python package, facilitates network construction from both expression matrix files and AnnData objects.

### 2.1 Pre-processing count data

The first step is to *log_2_* normalize the count matrices. By log-transforming the values, we model proportional changes rather than additive changes and we help the model focus on the biologically relevant differences rather than the extreme values. The general recommendation is to ensure that the data lies in the range of the function we are using to approximate it, in our case a neural network with sigmoid activation function on the output layer. Then, we normalized the values to the [0,1] range by dividing by the max value of the row that each value belongs.

### 2.2 Dimensionality reduction using variational autoencoders and hyperparameters

To compress the high-dimensional expression matrices into lower-dimensional latent spaces, we make use of VAEs. Using VAEs, the latent representations are regularized by minimizing the regularization loss, implemented with the Kullback–Leibler (KL) divergence between the latent and a prior, in this case a normal distribution with mean 1 and 0.1 standard deviation. The VAE loss is composed by the reconstruction and regularization losses weighted sum, where the weights are set to 0.9 and 0.1 respectively, obtained through hyperparameter tuning. Regarding the VAEs architecture, VAE performance was evaluated with different layers. We use a single hidden layer in FAVA. In all cases, we used the Rectified Linear Unit function (Nair and Hinton 2010) for all layers, except for the mean (μ) and var (σ) encoding layers and the last layer in the decoder. In the output layer, considering the 0–1 scaling of the inputs, a sigmoid function was used to generate values between 0 and 1, whereas linear activation was set for the mu and sigma layers. To train the VAE, we used the Adam optimizer with a learning rate of 10^−3^. The VAE model was implemented in Keras (https://keras.io/).

### 2.3 Pearson correlation coefficient pairwise on the latent space

Having produced a regularized latent space that follows Gaussian distribution from the VAE, we calculate all pairwise PCCs between proteins in the latent space. That outputs a list of protein pairs with an assigned score, showing the proximity of the two proteins in the latent space. Based on this score, we sort all protein pairs and create a ranked list, in which numbers closer to 1 represent higher proximity in the latent space and thus, expression similarity. Finally, we benchmark the resulting ranked list against the KEGG database (Kanehisa *et al.* 2021) to quantify how well the predicted interactions agree with what is known.

### 2.4 Functional association scoring in the latent space

Having produced a regularized latent space that follows Gaussian distribution from the VAE, we calculate all pairwise PCCs between proteins in the latent space. That outputs a list of protein pairs with an assigned score, showing the similarity of the two proteins in the latent space. Based on this score, we sort all protein pairs and create a ranked list, in which numbers closer to 1 represent higher similarity in the latent space and thus, expression similarity.

### 2.5 Other methods

#### 2.5.1 scLink

To create the network of the small dataset using the scLink method, we followed the pipeline described here (https://cran.r-project.org/web/packages/scLink/scLink.pdf).

#### 2.5.2 hdWGCNA

To create the networks using hdWGCNA, we first had to analyze the data using the Seurat (Hao *et al.* 2020) pipeline. Thus, we followed the tutorial provided by Seurat here (https://satijalab.org/seurat/articles/pbmc3k_tutorial.html). That creates a Seurat object (seurat_obj) which can be utilized further by hdWGCNA. Afterward, we followed the recommendations of the authors to construct the network as described in their tutorial here (https://smorabit.github.io/hdWGCNA/articles/basic_tutorial.html).

#### 2.5.3 Pearson correlation coefficient

We calculated all-against-all PCC on the same log-transformed counts matrices used as input for FAVA. This was done in order to have a fair comparison with FAVA.

For more details, see Supplementary Material S1A.

### 2.6 Datasets

FAVA, scLink, hdWGCNA, and PCC were applied on the datasets below to create functional association networks and then benchmarked against ground-truth GRNs:

**Human Glioblastoma Multiforme:** Approximately 1500 cells from a 71-year-old male, using the Chromium Next GEM Single Cell 3ʹ Kits v3.1. Sequenced on Illumina NovaSeq 6000.

**Human Squamous Cell Lung Carcinoma:** Around 2500 cells from Stage III lung carcinoma. Generated with Chromium Single Cell 5' Kits and sequenced on Illumina NovaSeq 6000.

**Small PBMCs Dataset:** 2700 PBMC, sequenced using the Chromium kit from 10x Genomics.

**Human Pancreatic Tumor:** About 6500 cells, sequenced with Chromium Single Cell 5'v2 Kits on Illumina NovaSeq.

**Large PBMCs Dataset:** 30 478 cells sequenced with the Evercode™ WT kit. Data from Parse Biosciences.

**Human Protein Atlas:** Single-cell RNA-seq data from 566 109 cells, profiling 19 670 genes. The matrix gives read counts for 19 670 human protein-coding genes in 566 109 individual cells grouped into 192 cell type clusters. Information about the external datasets and processing of the data is described in detail in Karlsson *et al.* (2021).

**PRIDE EMBL-EBI Proteomics Dataset:** Analysis of 633 human proteomics experiments, with a total of 32 546 runs, reanalyzed them using ionbot (Degroeve *et al.* 2021) with an FDR threshold of 0.01 (Käll *et al.* 2008), resulting in a total of 154 885 151 peptide spectrum matches for 18 846 proteins. For the full list of projects, runs, and general statistics of the search see Supplementary Material in Zenodo (doi: 10.5281/zenodo.6798182).

**Simulated datasets:** SERGIO and scMultiSim: Used to create single-cell expression matrices based on Gene Regulatory Networks (GRNs).

For more details, see Supplementary Material S1B and S1C.

### 2.7 Benchmark of functional associations

**KEGG Database (Release 95)**: Protein pairs from various methods are mapped to KEGG maps. Pairs found in a map are true positives (TP), while those not in any map are false positives (FP). Unmappable pairs are excluded. Results are visualized by plotting cumulative TP against FP counts.

**Reactome Pathways** (Gillespie *et al.* 2022) **(Release 85)**: Similar to KEGG, protein pairs are mapped to Reactome pathways. Inclusion in a pathway denotes a TP, and absence a FP. Unmappable pairs are disregarded.

**Complex Portal** (Meldal *et al.* 2019) **(June 2023)**: Protein pairs are matched with Complex Portal entries. Those included in an entry are TPs, and those absent are FPs. Unmappable pairs are excluded.

**BioGRID** (Oughtred *et al.* 2021) **(Release 4.4.217)**: The BioGRID interaction network serves as ground truth. Predicted pairs in BioGRID are TPs, while absent ones are FPs.**hu.MAP 2.0**: The hu.MAP 2.0 database is used for validation. Pairs present in the database are TPs, and those not found are FPs.

**BioPlex 3.0** (Huttlin *et al.* 2021) **Interactions (293 T Cells)**: The BioPlex interactome is used for validation. Pairs present in the interactome are TPs, and those not found are FPs.

For more details, see Supplementary Material S1D.

### 2.8 Score calibration and combination of FAVA networks from atlases

To combine the two networks, we decided to apply the pipeline used in the STRING database. Thus, we first convert the PCC scores from FAVA from each dataset into posterior probabilities of being on the same KEGG map given the PCC (Fig. 1). We do this based on the benchmark results described above, by first plotting the local precision within a sliding window (y = TP/(TP + FP)) as a function of the average PCC within the window (x). We do this separately for scRNA-seq and bulk proteomics data and fit the following calibration function to each by minimizing the squared error using simplex optimization:

y=a0+a1*x+a2/(1+exp (a3*(x−a4))),

where a0 through a4 are the parameters that are optimized to fit the points in the plot. Once fitted, we use the resulting calibration curves to convert all PCCs from each dataset to probabilities. As the sigmoid function is a monotonic function, it purely converts PCCs into posterior probabilities that are easier to interpret, but does not reorder the gene pairs. When a pair is supported by both scRNA-seq and bulk proteomics data, the two probabilities are combined.

### 2.9 Definition of understudied proteins

To define “understudied proteins”, we use the knowledge-based classification for human proteins, “Target Development Level” (TDL), as proposed by the National Institutes of Health (NIH) funded consortium “Illuminating the Druggable Genome” (IDG) (Oprea 2019). Specifically, IDG provides information on human proteins (targets), dividing them into four levels for drug development: “Tclin” are drug targets with known mechanisms of action approved by DrugCentral; “Tchem” are targets selected from ChEMBL/DrugCentral or manually curated from other sources; “Tbio” are biologically characterized targets without known drug or small molecule activities that meet specified activity thresholds; and “Tdark” are “understudied proteins”, or targets with little known information and not meeting the drug/small molecule activity criteria (Sheils *et al.* 2020). In our analysis, we used the list of 6000 proteins that have been designated as Tdark (“dark” targets) by IDG (Sheils *et al.* 2021), downloaded in January 2022.

## 3 Results

We have developed a novel method for construction of functional association networks from huge omics datasets with high data sparseness and redundancy. The method makes use of VAEs to perform dimensionality reduction and subsequently scores functional associations in the latent space.

FAVA utilizes scRNA-seq data from the Human Protein Atlas (Uhlén *et al.* 2015) and bulk proteomics data from the PRIDE database (Perez-Riverol *et al.* 2019) as inputs to train two separate VAEs (Fig. 1, top and main box). VAEs are then used to learn the distribution of the input data and create a meaningful low-dimensional representation (i.e. the latent space). Lastly, all-against-all PCCs are calculated in the latent space to quantify similarity in expression between any two genes/proteins. The resulting adjacency matrix is directly translated to a weighted functional association network, one for each data type. To combine the two networks, we first convert the Pearson scores into posterior probabilities of belonging to the same biological pathway (KEGG map) by fitting a calibration function (Fig. 1, equation plot). If an interaction is supported by both scRNA-seq and bulk proteomics data, the two probabilities are combined. The resulting combined network (Fig. 1, bottom) thus takes advantage of the complementary nature of the two datasets and is enriched for understudied proteins. While the network provided in this paper is based on these specific datasets, the method is applicable to scRNA-seq datasets in general.

The method is implemented in Python and makes use of the Keras deep-learning framework for training VAEs. The software is available via PyPI as ‘favapy’ and is distributed under the MIT open source license. It can be used either directly from the command line, taking an expression matrix file as argument, or as a module in Python scripts. In the Python module, the input can be either a matrix with counts or abundance values from any omics dataset or an AnnData (Virshup *et al.* 2021) object. The latter is a generic class for handling annotated data matrices making it simple to perform functional association analysis from scRNA-seq data using popular pipelines.

### 3.1 FAVA outperforms state-of-the-art methods for functional association network construction from individual scRNA-seq datasets

To assess the performance of FAVA on scRNA-seq data, we tested the method on five diverse datasets: 1653 (1.5k) human glioblastoma multiforme cells (obtained via 10x Genomics), 2616 (2.5k) human squamous cell lung carcinoma cells (using Chromium X chemistry), 2700 (3k) single PBMC (sequenced with the Chromium kit from 10x Genomics), 6647 (6.5k) human pancreatic tumor cells (isolated with the Chromium Nuclei Isolation Kit), and 30 478 (30k) PBMC (sequenced with the Evercode™ WT kit from Parse Biosciences). For more details on the datasets, see Section 2. These datasets were selected to cover a range of sizes, origins, and chemistries; moreover, some of them have been used as benchmarks in other studies (Wolf *et al.* 2018, Hao *et al.* 2020). The performance of the resulting functional association networks was evaluated against six gold standard benchmarks, namely manually annotated pathways from KEGG (Kanehisa *et al.* 2021) and Reactome (Gillespie *et al.* 2022), physical protein interactions from Complex Portal (Combe *et al.* 2017, Meldal *et al.* 2019), BioGRID (Oughtred *et al.* 2021), hu.MAP 2.0 (Drew, Wallingford and Marcotte 2021), and the cell-line-specific interaction network BioPlex 3.0 (Huttlin *et al.* 2021) that resulted from affinity purification of half the human proteome (10 128 proteins) in 293 T cells and yielded 118 162 interactions among 14 586 proteins. For comparison, we also generated networks using three other functional association methods that are either commonly used or specifically designed for scRNA-seq data, namely PCC, hdWGCNA (Morabito *et al.* 2022), and scLink (Li and Li 2021).

**Figure 2.**
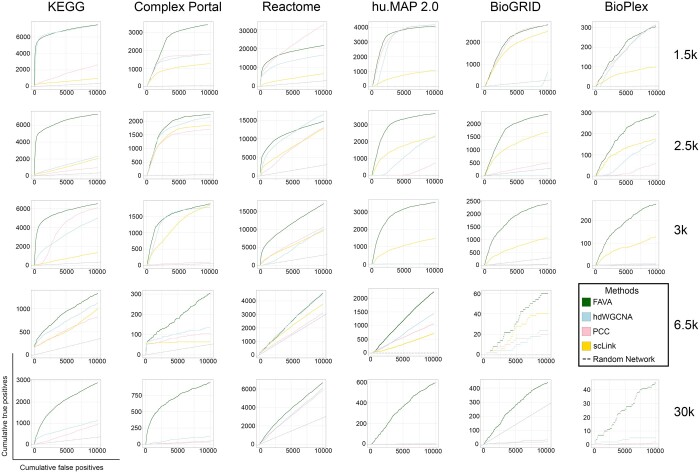
Comparison of the performance of FAVA and the other gene network inference methods on individual scRNA-seq expression data. The plots show method accuracy on functional association networks based on different benchmark sets, namely KEGG, Complex Portal, Reactome, hu. MAP 2.0, and BioGRID. Each dataset represents different cell sizes: 1.5k (approximately 1500 cells of human glioblastoma multiforme obtained through 10x Genomics), 2.5k (approximately 2500 cells from human squamous cell lung carcinoma using Chromium X chemistry), 3k (2700 single PBMC sequenced with the Chromium kit from 10x Genomics), 6.5k (approximately 6500 cells from human pancreatic tumor isolated with Chromium Nuclei Isolation Kit), and 30k (30 478 PBMC v2 sequenced with the Evercode™ WT kit from Parse Biosciences). As it was not possible to run scLink on the large 30k dataset, this method is only included in the plots for the four smaller datasets. For KEGG and Reactome pathways, a true positive means that both genes/proteins in a pair are found in the same KEGG/Reactome map, and a false positive is when both genes/proteins are found in different KEGG or Reactome maps, respectively. For Complex Portal, a true positive means that both genes/proteins in a pair are found in the same complex, and a false positive is when both genes/proteins are found in different complexes. For BioGRID, hu.MAP 2.0 and BioPlex, a true positive is a pair of genes/proteins that is also present in the BioGRID/hu.MAP 2.0/BioPlex interactome, while a false positive is a pair not present in BioGRID or hu.MAP 2.0 or BioPlex, respectively.

Figure 2 shows that FAVA outperforms the other methods by a wide margin on all the datasets and with respect to the six gold standards. For a simplified interpretation of the curves, we here focus on the true positive/false positive (TP/FP) ratios in the top 10 000 interactions from each method for two of the six of the gold standards, namely KEGG and BioGRID. On the small dataset, FAVA gave a TP/FP ratio of 3.9 (4588/1168) according to the KEGG benchmark (Fig. 2a), which is 3.9 times better than the second best method, scLink (1878/1860 = 1.0). For the BioGRID benchmark, the corresponding ratios are 0.29 (2266/7734) and 0.1 (1014/8986) for FAVA and scLink, respectively (Fig. 2b). On the large dataset, which was too large for scLink to process, FAVA also produces the best results with TP/FP ratios of 0.55 (988/1800) and 0.04 (430/9570) for the KEGG and BioGRID benchmarks, respectively (Fig. 2c and d). By comparison, the two other methods (PCC, hdWGCNA) barely manage to perform better than random. Results are similar across all gold standards as showcased in Fig. 2.

We thus conclude that the functional association networks produced by FAVA based on scRNA-seq data are better than the corresponding networks from other methods at predicting which proteins function in the same pathway as well as which proteins bind to each other. While neither gold standard is a perfect ground truth for evaluating functional association networks, the consistency of the results on six gold standards and five datasets is reassuring.

Precision curves, for the pathway databases KEGG and Reactome, have been included in the Supplementary Material (see Supplementary Fig. S2). We did not include hu.MAP 2.0, BioGRID, and Complex Portal in these plots, as they primarily consist of physical interactions, whereas our focus is on functional association networks. Counting all non-physical interactions predicted by FAVA or other functional association methods as false positives would inherently result in dramatic underestimation of the precision of all methods.

These findings emphasize two key observations. First, regardless of the dataset size or benchmark sets used, FAVA consistently outperforms other methods (as evidenced also by its performance on simulated datasets shown in Supplementary Fig. S3). Second, the baseline method, PCC, emerges as the second-best performer across various datasets and benchmark sets.

To further investigate the impact of the dataset size on network quality, we subdivided the HPA single-cell atlas into individual tissues and ran FAVA on each. Intriguingly, our findings challenge the notion that a larger number of cells inherently leads to improved performance (Supplementary Fig. S4). Specifically, we observed that networks derived from a smaller number of cells exhibited superior performance compared to some others constructed from larger cell populations. This observation underscores the fact that data quality cannot be solely attributed to the quantity of cells, but rather, various dataset-specific characteristics play a substantial role in determining analysis accuracy.

Finally, we assessed the potential for the FAVA algorithm to exhibit overfitting. In FAVA, separate VAEs are trained to learn the distribution of individual datasets in an unsupervised manner, and the trained models are not applied to unseen data. As no fitting takes place on benchmark datasets, any overfitting of the VAEs to the input data should thus not result in the good performance that we observe. Rather, overfitting should result in a less meaningful latent space and consequently worse performance. To nonetheless evaluate if overfitting happens, we chose another scRNA-seq dataset [GSE75748 (Chu *et al.* 2016)] and randomly split it into a training and a test set, consisting of 67% and 33% of the original dataset, respectively. We trained the VAE only on the training set and tried to predict interactions from both the training and the test set. Similar performance was obtained for both training and test sets (Supplementary Fig. S5), suggesting that our models do not overfit the data and that they are even able to generalize to unseen data, although such generalization may not be necessary for the specific use cases presented. The accuracy of these networks was evaluated using KEGG.

### 3.2 Application of FAVA to ∼0.5M single cells and ∼32k proteomics studies

To assess the ability of FAVA to process huge, diverse omics datasets and infer novel functional associations, we applied the method to the single-cell dataset from the Human Protein Atlas and to bulk proteomics data on human samples from the PRIDE database. We evaluated the quality of the resulting functional association networks by benchmarking them against pathways from the KEGG database (Section 2).

**Figure 3.**
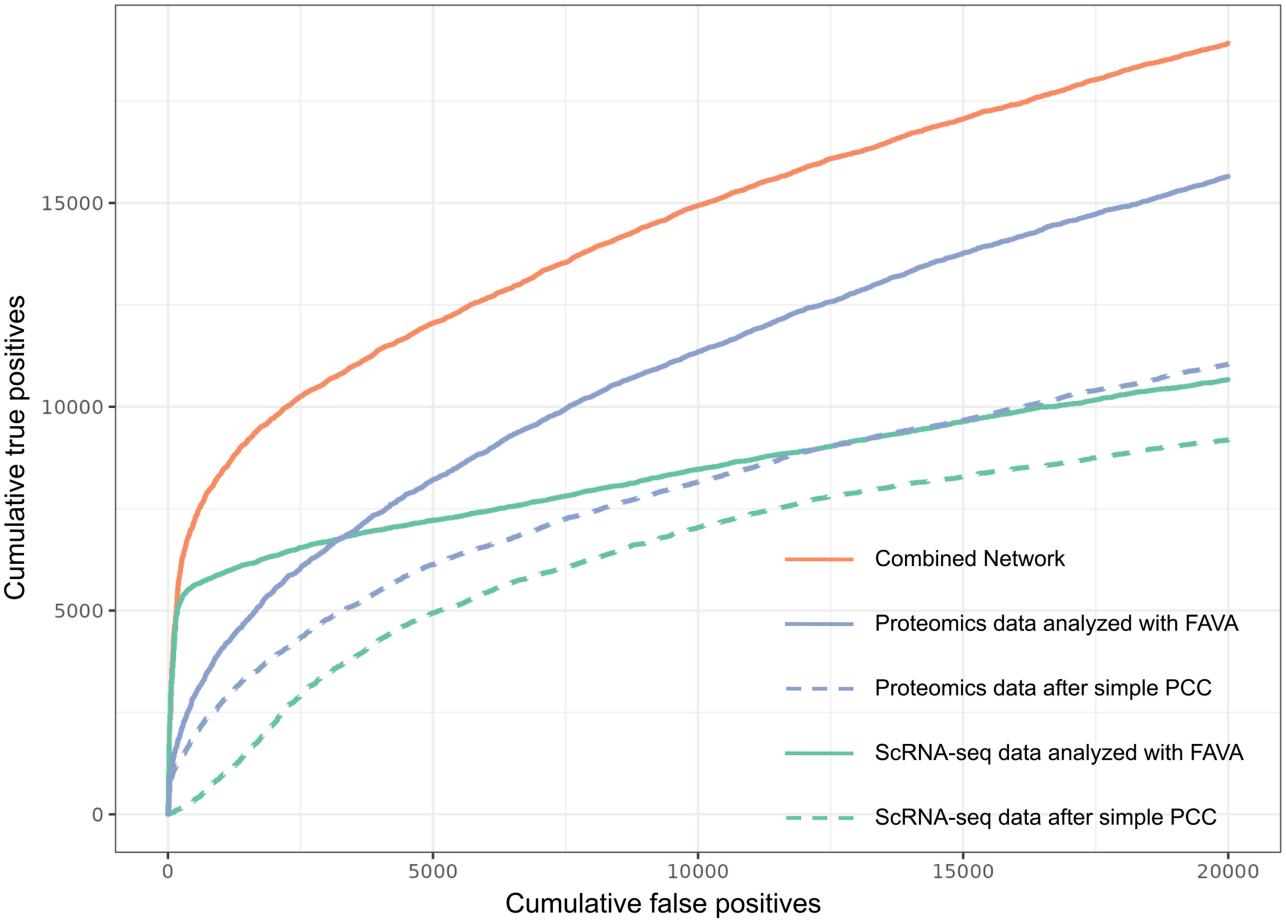
Comparison of FAVA’s results against networks obtained by calculating PCC. The plots show how many known interactions can be predicted according to the KEGG database with the two different methods. True positive is a pair of proteins when both proteins are found in the same KEGG map. False positive is a pair of proteins when the two proteins are found in different maps of KEGG. Blue continuous line: benchmark network from bulk proteomics data after applying FAVA; blue dashed line: benchmark network from bulk proteomics data after applying traditional PCC; green continuous line: benchmark network from scRNA-seq data after applying FAVA; green dashed line: benchmark network from scRNA-seq data after applying traditional PCC; orange: resulting network after combining scRNA-seq and proteomics networks coming from FAVA, using KEGG for score calibration (Section 2). We also evaluated the performance of Spearman correlation on the bulk proteomics data (see Supplementary Fig. S6).

Here we show that the network derived from scRNA-seq data (Fig. 3, green continuous line) captures a very large number of high-confidence interactions in KEGG; it is possible to obtain more than 5000 TPs with less than 500 FPs, corresponding to a precision of more than 90%. The proteomics-based network (Fig. 3, blue continuous line), by contrast, provides fewer high-confidence interactions but more interactions at lower confidence, with the two methods performing on par at ∼3000 FPs. Comparing the FAVA results to those obtained from PCC on the single-cell and bulk proteomics data (Fig. 3, dashed lines) shows that FAVA performs better by a wide margin on both types of data, and especially on scRNA-seq. We did not include scLink and hdWGCNA in this comparison, because the former is unable to handle such large datasets, and both are designed specifically for scRNA-seq data and are thus not applicable to the proteomics dataset.

### 3.3 Combined network from scRNA-seq and bulk proteomics data

Given the complementary nature of the networks based on scRNA-seq and bulk proteomics data individually, we decided to combine them into a single network. As the PCC scores from FAVA cannot be assumed to be directly comparable across the two networks, we converted them to probabilistic scores by calibrating both on the KEGG benchmark (Section 2). These calibrated scores were then combined to produce a single network based on scRNA-seq as well as bulk proteomics data. Figure 3 shows the performance of the combined network (orange continuous line) derived from both the scRNA-seq and the proteomics networks (green and blue continuous lines, respectively). As should be expected, the combined network shows better agreement with KEGG than either of the individual networks that it was created from. Crucially, we do not consider only the intersection of the networks, which could result in a sparse network with reduced sensitivity (recall); we consider all interactions from both types of data (i.e. the union), combining scores for interactions supported by both types of data.

As was the case for the individual networks, the benchmark against KEGG can also be used to assign probabilistic scores to the interactions in the combined network. At a 15% confidence cutoff (corresponding to the low-confidence cutoff of STRING), the combined network consists of a total of 511 048 associations for 16 790 proteins. The network can be further filtered to obtain higher confidence networks, depending on the concrete use case; in case of proteins with many associations, one will generally want to focus on the highest scoring ones. The combined network has 52 953 associations between 8191 proteins at the medium confidence cutoff (40%), and even at the high confidence cutoff (70%), it provides 24 182 interactions among 4166 proteins. The complete combined network, comprising connections with a confidence score of 15% or higher, can be downloaded in its entirety (doi: 10.5281/zenodo.6803472).

### 3.4 Associations for understudied proteins

Given the nature of the data that the combined network is based on, it should be free of the inherent literature bias, which many other protein networks suffer from. Additionally, the network incorporates numerous high-confidence interactions, rendering it highly suitable for the exploration and understanding of the functions associated with understudied proteins.

**Figure 4.**
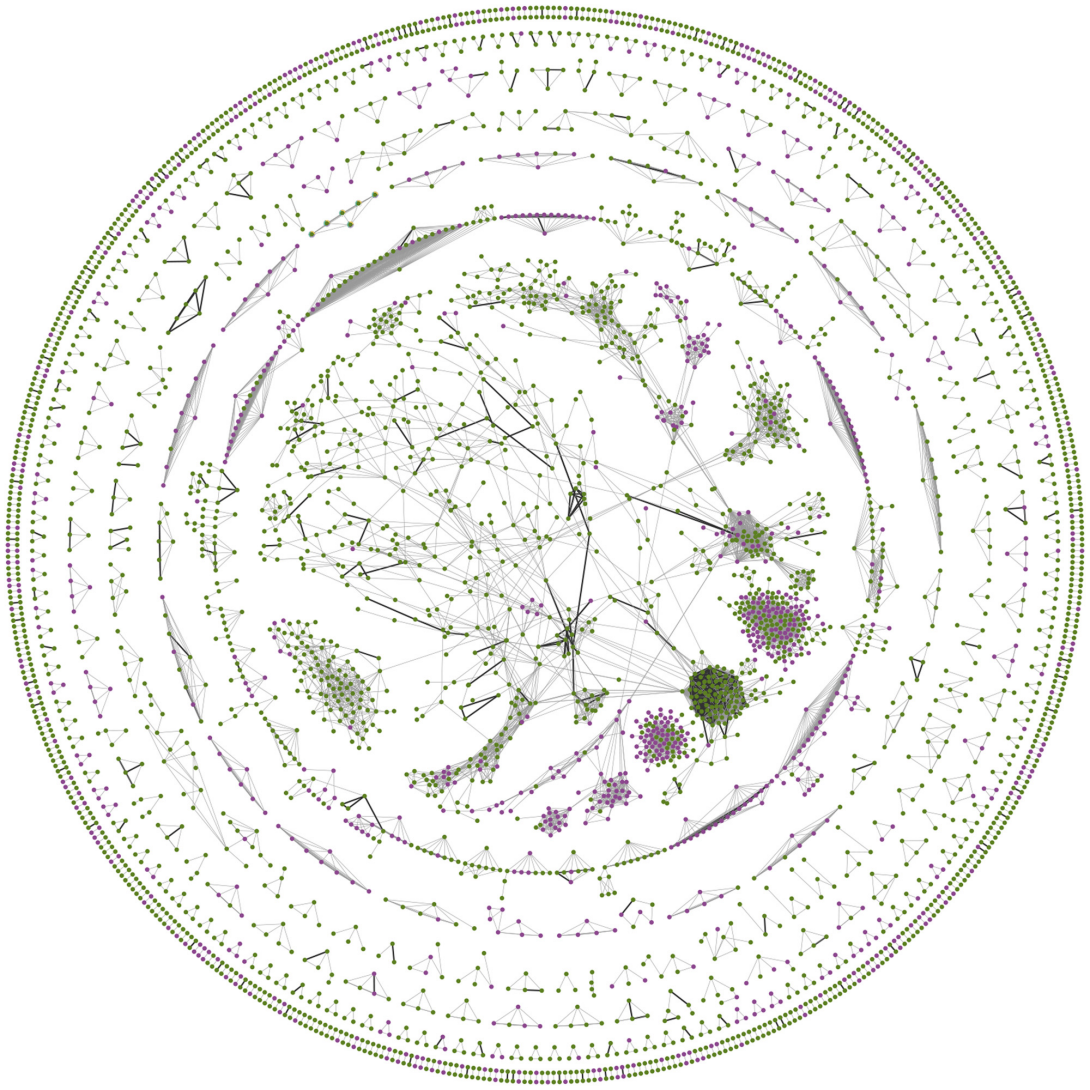
Understudied proteins in the combined FAVA network. The figure shows all functional associations with a confidence score of at least 70%, based on FAVA analysis of both the scRNA-seq and proteomics data. Understudied proteins according to IDG are highlighted in purple. The edges in the network which are physical interactions according to hu.MAP 2.0 are highlighted with darker, thicker edges. The visualization of the network was made in Cytoscape (Shannon *et al.* 2003), and the analysis for the IDG target classification was retrieved using Cytoscape stringApp (Doncheva *et al.* 2019).

As described in Section 2, our analysis focused on 6000 proteins classified as Tdark (“understudied proteins”) by IDG. Looking these up in the combined FAVA network, based on both scRNA-seq and bulk proteomics data, revealed 4210 predicted interactions between 1039 of the understudied proteins (Fig. 4, purple nodes) and 611 other, better studied proteins (Tclin) with at least 70% confidence. Next, we compared this network to hu.MAP 2.0 (Drew *et al.* 2021) to evaluate both the agreement and the complementarity of the two networks. Since hu.MAP 2.0 is a physical interaction network, it should contain only a subset of the interactions from the combined FAVA network. In Supplementary Fig. S7, we report the precision and recall calculations of our network on hu.MAP 2.0 edges, showing the agreement between the two networks. Furthermore, comparing the two networks, both at 70% confidence, we found that they agree on 1284 edges (Fig. 4, dark gray edges) for 520 nodes, which confirms the high quality of both networks and shows that the FAVA network includes physical interactions among the functional associations. Focusing on the understudied proteins, 49 of the 1039 that are found in the FAVA network are also among the 648 that hu.MAP 2.0 provides physical interactions for (see Supplementary Fig. S8 for a network with visible labels). This shows that while there is good agreement between the two networks, they are also highly complementary, illustrating that FAVA provides much needed additional information about different understudied proteins.

To evaluate the novelty of the interactions discovered by FAVA, we checked how many of the FAVA edges can be found in the STRING v11.5 and hu. MAP 2.0 databases. Using multiple score cutoffs for STRING, we analyzed the overlapping edges and found that the FAVA network contains 2747 high-scoring interactions for understudied proteins (Tdark) that are not present in the STRING network at all, and only two of these are found in hu.MAP 2.0 (Supplementary Fig. S9). This highlights the value of incorporating functional association analysis and demonstrates the ability of FAVA to uncover novel interactions, complementing existing experimental and literature knowledge.

### 3.5 Clustering and functional enrichment analysis

To assess whether functional modules can be detected in the FAVA network, we first clustered the network using the MCL algorithm (inflation value 1.5) (Meldal *et al.* 2019). This resulted in a network with 341 clusters, 130 of which comprised five or more nodes, and were further analyzed. Next, we used Cytoscape stringApp 2.0 (Doncheva *et al.* 2019) to perform enrichment analysis on the clustered network (Cytoscape session in Supplementary Material S10).

**Figure 5.**
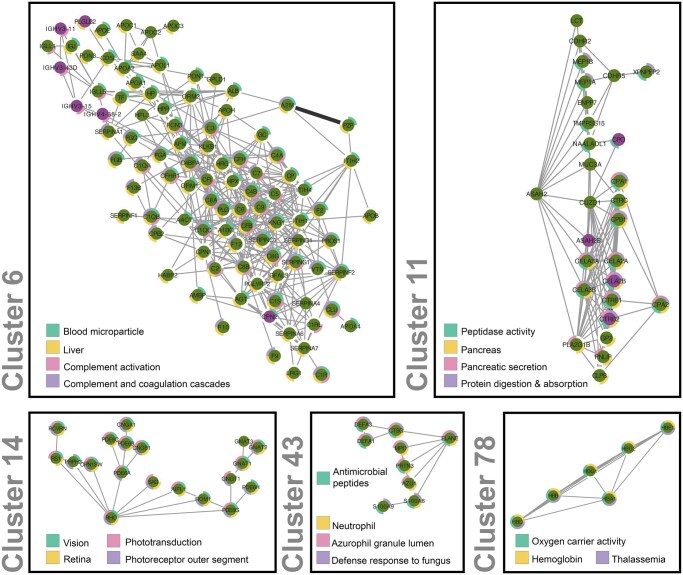
Clustering and functional enrichment analysis of the combined FAVA network. Five selected clusters with five or more nodes, derived utilizing the MCL algorithm, were visualized using Cytoscape (Shannon *et al.* 2003). The visual legend summarizes the enriched terms, depicted as quarters of colored rings around the protein nodes of each cluster. The clusters represent different functions and pathways Clusters 6 and 11 also illustrate how FAVA is able to link understudied proteins (Tdark; purple) to well-annotated ones (green).

The full enrichment results are available in Supplementary Table S1, Sheet 1. To functionally characterize the clusters, we manually selected highly significant terms from the following seven categories: namely Pathways, UniProt Keywords, Cellular Compartments, Tissues, Biological Processes, Molecular Functions and Diseases. Five selected clusters are shown in Fig. 5, with colored rings around the nodes to indicate the most descriptive functionally enriched terms for each cluster. Results for all clusters can be found in Supplementary Table S1, Sheet 2.

## 4 Discussion

Here we introduced FAVA, a new method to build functional association networks from omics data using VAEs. FAVA takes an expression matrix from, e.g. scRNA-seq or proteomics experiments as input and uses VAEs to create a low-dimensional representation of it, known as the latent space. It then produces a weighted functional association network by calculating PCCs in the latent space. To evaluate the ability of FAVA to handle sparse and redundant data, we have tested it on two scRNA-seq datasets of very different size and compared its performance to that of tools designed or commonly used for the same purpose, namely scLink, hdWGCNA and PCC. We evaluated the methods on six complementary benchmark sets, namely, pathways from KEGG and Reactome, complexes from Complex Portal, physical protein interactions from BioGRID and hu.MAP 2.0 and a cell-line-specific interaction network, BioPlex 3.0. Overall, FAVA demonstrated the best performance.

FAVA is an efficient, unsupervised, stand-alone method, which does not rely on the results from other approaches, as the hdWGCNA does. Additionally, it considers the expression count of all genes, while scLink only takes into consideration the expression counts of certain highly expressed genes. With that, FAVA avoids the accumulation of “rich-get-richer” genes/proteins and produces networks that contain both studied and understudied genes/proteins, which is a major issue in the field. Compared to PCC, FAVA is at least nine times faster (1 day ≃ 9 days) and is better at accurately identifying established patterns in the data.

The efficiency of FAVA allows the method to be applied not only to individual studies, but also to large expression atlases. This versatility of FAVA allows the construction of functional association networks from vast amounts of data of different types. As illustrated in Fig. 4, FAVA generates functional association networks from scRNA-seq and bulk proteomics data and outperforms conventional functional association methods. The combination of these networks offers new perspectives on uncharacterized proteins. This highlights the ability of the method to handle data beyond scRNA-seq. Overall, the efficiency of FAVA makes it a powerful and flexible approach for gene expression studies.

FAVA also achieves higher accuracy in gene network construction on simulated datasets, as presented on Supplementary Fig. S3. However, we remain skeptical about these results, because the simulation algorithms used were not designed for the same purpose as the methods discussed. Indeed, we utilized simulation algorithms that create expression datasets based on GRNs. Then we evaluated the performances of FAVA, scLink, hdWGCNA, and PCC, methods developed for building functional association networks, regarding their accuracy on GRN predictions. We concluded that while none of the methods had accuracy suitable for this task, FAVA performed better than the other methods.

## 5 Conclusions

In this work, we have shown that VAEs can be used to model single-cell RNA-seq and bulk proteomics data for prediction of functional associations. We have applied this method, which we call FAVA, to large compendiums of scRNA-seq data as well as bulk proteomics data. The results show that FAVA scales to such large datasets and that the resulting networks are considerably more reliable than those obtained from traditional functional association methods to the same data. We have moreover shown that combining FAVA results from both data types provides an even more comprehensive network and that this network can be used to associate understudied proteins with better studied ones, thereby providing hints to their possible functions. We make these networks publicly available along with the Python implementation of FAVA, which can be installed as the PyPI package ‘favapy’.

## Supplementary Material

btae010_Supplementary_Data

## Data Availability

The data underlying this article are available in The Network: https://doi.org/10.5281/zenodo.6803472 and The PRIDE database analysis: the full list of projects, runs, and general statistics about the analysis of the data in the PRIDE database: https://doi.org/10.5281/zenodo.6798182.
