# Supplementary material for: FAVA: high-quality functional association networks inferred from scRNA-seq and proteomics data
Source: Bioinformatics. 2024 Jan 8;40(2):btae010. doi: 10.1093/bioinformatics/btae010 (PMC10868155; doi:10.1093/bioinformatics/btae010)
Supplement: btae010_Supplementary_Data [file btae010_supplementary_data.zip › FAVA_Bioinformatics_SupplementaryMaterial.pdf]

Supplementary material

S1A. Other Methods

scLink

To create the network of the small dataset using the scLink method, we followed the pipeline described here. Thus, we normalized the data with the package's function and filtered for the 500 most highly expressed genes, as recommended by the authors: `sclink_norm(count, scale.factor=1e+06, filter.genes = TRUE, n = 500)`. Then we calculated the correlation matrix with the function: `sclink_cor(expr = count.norm)`. We were not able to process the large dataset on a server with 180GB RAM.

hdWGCNA

To create the networks using hdWGCNA, we first had to analyze the data using the Seurat pipeline. Thus, we followed the tutorial provided by Seurat here. That creates a Seurat object (seurat_obj) which can be utilized further by hdWGCNA. Afterwards, we followed the recommendations of the authors to construct the network as described in their tutorial here. Therefore, we ran in R the following commands:

```
seurat_obj = SetupForWGCNA(seurat_obj, gene_select = "fraction", fraction = 0.05)
seurat_obj = SetDatExpr(seurat_obj, assay = 'RNA', slot = 'data')
seurat_obj = TestSoftPowers(seurat_obj, networkType = 'unsigned')
```

Based on the results coming from the `GetPowerTable(seurat_obj)` function, we selected the soft_power parameter (4 for the small dataset and 7 for the large dataset). Final step was to construct the network:

```
seurat_obj = ConstructNetwork(seurat_obj, soft_power = soft_power, setDatExpr = FALSE, overwrite_tom = TRUE, networkType = 'unsigned')
```

S1B. Datasets

Human Glioblastoma Multiforme (~1,500 cells)

This dataset consists of 2,000 sorted cells from Human Glioblastoma Multiforme, prepared using the Chromium Next GEM Single Cell 3' Reagent Kits v3.1. The cells were obtained from a 71-year-old male donor and provided by 10x Genomics through Discovery Life Sciences. The libraries were sequenced on an Illumina NovaSeq 6000, with an average sequencing depth of 72,259 reads per cell. The analysis was performed using Cell Ranger 6.0.0 with the parameter "--expect-cells=2000". After filtering the number of cells decreased to 1,653.

Human Squamous Cell Lung Carcinoma (~2,500 cells)

This dataset is part of the Alternative transcript isoform detection with single cell and spatial resolution. It includes cryopreserved, dissociated tumor cells from Stage III squamous cell lung carcinoma (lung cancer DTCs) obtained from Discovery Life Sciences. The dataset consists of 5' Single Cell Gene Expression Libraries generated from approximately 4,000 cells, with 2,616 cells successfully recovered. The libraries were prepared following the

Chromium Single Cell 5' Reagent Kits User Guide (v2 Chemistry Dual Index) and sequenced on an Illumina NovaSeq 6000, achieving an average read depth of around 25,000 reads per cell.

Small PBMCs dataset (3,000 cells)

This dataset consists of 2,700 single Peripheral Blood Mononuclear Cells (PBMC) that were sequenced using Chromium kit from 10x Genomics. We downloaded the counts matrix for this freely available dataset from 10X Genomics:

Human Pancreatic Tumor (~6,500 cells)

This dataset comprises 5,000 human pancreatic tumor cells isolated using the Chromium Nuclei Isolation Kit. The tumor tissue, obtained from Discovery Life Sciences, was processed following the Chromium Nuclei Isolation Reagent Kits Sample Prep User Guide. Gene expression libraries were prepared using the Chromium Single Cell 5'v2 Reagent Kits and sequenced on an Illumina NovaSeq instrument with a target of 20,000 reads per cell. The data were processed using Cell Ranger 7.0.0, resulting in the detection of 6,647 cells, with a median of 1,776 genes and 3,465 UMIs per cell. The sequencing depth averaged 56,169 reads per cell in a paired-end, dual indexing configuration.

Large PBMCs dataset (~30,000 cells)

This larger dataset of 30,478 Peripheral Blood Mononuclear Cells (PBMC v2) was sequenced after applying the Evercode™ WT kit. We downloaded the counts matrix for this freely available dataset from Parse Biosciences:
www.parsebiosciences.com/datasets

Human Protein Atlas – Single-cell RNA-seq read-count data

We obtained the single-cell dataset from the Human Protein Atlas (https://www.proteinatlas.org/humanproteome/single+cell+type), a public resource that provides transcriptomics and spatial antibody-based proteomics profiling of human tissues. Their single-cell transcriptomics atlas combines data from 26 datasets. The matrix gives read counts for 19,670 human protein-coding genes in 566,109 individual cells grouped into 192 cell type clusters. Information about the external datasets and processing of the data is described in detail in (36).

PRIDE EMBL-EBI – Proteomics dataset

We obtained our proteomics dataset from The PRoteomics IDEntifications (PRIDE - https://www.ebi.ac.uk/pride/) database, the world's largest data repository of mass spectrometry-based proteomics data (46). Specifically, we used 633 human proteomics project experiments with a total of 32,546 runs and reanalyzed them using ionbot (37) with an FDR threshold of 0.01 (38), resulting in a total of 154,885,151 peptide spectrum matches for 18,846 proteins. For the full list of projects, runs, and general statistics of the search see supplementary material in Zenodo (doi: 10.5281/zenodo.6798182).

S1C. Simulated Datasets

To benchmark the functional association network methods on simulated data, we used two recent simulators, SERGIO and scMultiSim, to create single-cell expression matrices based

on GRNs. We then used FAVA, scLink, hdWGCNA, and PCC to create functional association networks, and benchmarked these on the ground-truth GRNs.

scMultisim

To construct an expression matrix with scMultisim, we follow the pipeline described here: https://zhanglabgt.github.io/scMultiSim/vignettes/sim_new.nb.html. To allow for generation of a matrix with 2,000 cells and 1,000 genes we ran the following command: `options_ = list(rand.seed=0, GRN=GRN_params_100, num.cells=2000, diff.cif.fraction=0.8, intrinsic.noise=1).` The given GRN contains 1,432 interactions.

SERGIO

From SERGIO, we used the `SERGIO_noised_1200G_9T_300cPerT_dynamics_6_DS83` file, which is a simulated matrix populated with 300 cells and 1,200 genes and includes technical noise. The package also offers the equivalent GRN with 2,713 interactions, from which the simulated matrix is derived.

S1D. Benchmarks

KEGG

We benchmark the resulting ranked list against the KEGG database, identical to how functional associations are benchmarked in the STRING database (4), to quantify how well the predicted interactions agree with what is known. To do this, we first map the protein pairs from the methods to KEGG maps. If a KEGG map exists, which contains both proteins of a pair, the pair is counted as a true positive (TP). If both proteins can be mapped to KEGG, but there is no map containing both, the pair is counted as a false positive (FP). Pairs for which one or both proteins cannot be mapped to KEGG are disregarded for benchmarking purposes. Having defined which protein pairs are considered TPs and FPs, we plot the cumulative TP count as a function of the cumulative FP count for the sorted lists of pairs. Furthermore, we generate a plot that illustrates the precision in relation to the TP predictions for the sorted list of protein pairs obtained from the methods.

Reactome

Similar to the approach used in benchmarking with the KEGG database, Reactome pathways can be utilized to assess the concordance between predicted interactions and known functional associations. Protein pairs obtained from the prediction methods are mapped to the Reactome pathways. If a Reactome pathway contains both proteins of a pair, it is considered a true positive (TP). However, if both proteins can be mapped to Reactome but no pathway encompasses both, the pair is counted as a false positive (FP). Pairs where one or both proteins cannot be mapped to Reactome are excluded from the benchmarking analysis.

Complex Portal

We evaluate the performance of the ranked list by comparing it to the Complex Portal database. Protein pairs obtained from the methods are mapped to the entries in the Complex Portal. If a Complex Portal entry includes both proteins of a pair, it is considered a

true positive (TP). If both proteins can be mapped to the Complex Portal but no entry contains both, it is counted as a false positive (FP). Pairs with unmappable proteins are excluded. The evaluation is visualized through a plot showing the cumulative TP count against the cumulative FP count for the sorted lists of pairs. By benchmarking against the Complex Portal database, we assess how well the predicted interactions align with known information regarding physical protein complexes.

BioGRID

To measure how accurately the networks from the different methods correlate with BioGRID's experimental results, we considered the interaction network from BioGRID as our ground truth. If a predicted pair existed in BioGRID, it was labeled as a True Positive (TP). Conversely, if it was not present in BioGRID, it was labeled as a False Positive (FP).

hu.MAP 2.0

To evaluate the accuracy of the networks generated by different methods in correlation with experimental results, we utilized the hu.MAP 2.0 database as our benchmark. The hu.MAP 2.0 database provides a comprehensive resource for studying molecular interactions. In this evaluation, we considered the interaction network within hu.MAP 2.0 as our reference or ground truth. For each predicted protein pair, we checked if the pair existed in the hu.MAP 2.0 database. If a predicted pair was present in hu.MAP 2.0, it was labeled as a True Positive (TP), indicating a correct prediction. On the other hand, if a predicted pair was not found in hu.MAP 2.0, it was labeled as a False Positive (FP).

BioPlex 3.0

To assess the accuracy of networks created by various methods against experimental data, we used the BioPlex 3.0 database as our standard. BioPlex 3.0 serves as an extensive tool for exploring molecular interactions. In our assessment, the interaction network from BioPlex 3.0 was the reference or 'ground truth'. We verified the existence of each predicted protein pair in the BioPlex 3.0 database. A predicted pair found in BioPlex 3.0 was classified as a True Positive (TP), signifying an accurate prediction. Conversely, a predicted pair absent in BioPlex 3.0 was marked as a False Positive (FP).

S2. Precision curves of KEGG and Reactome

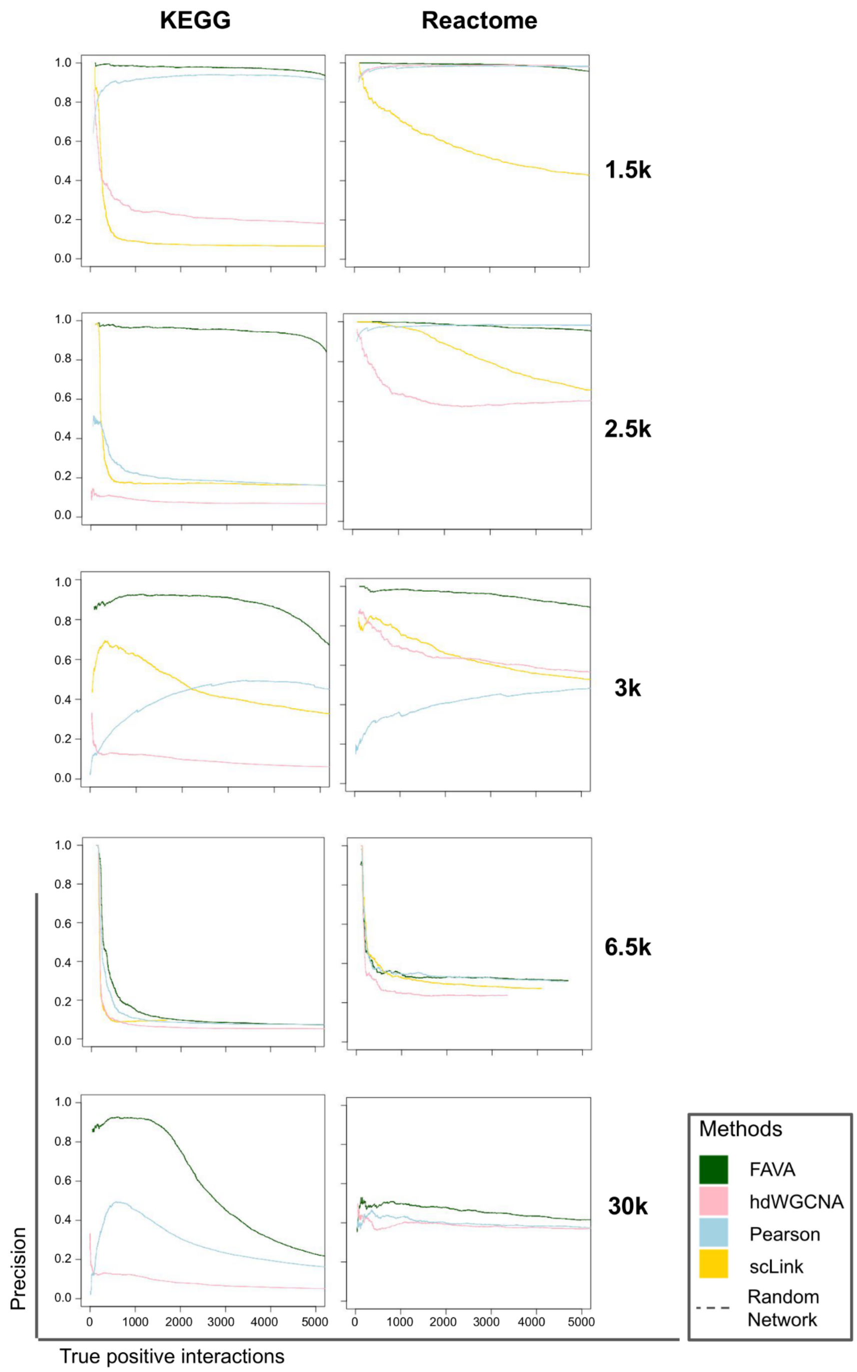

Figure S2. Precision curves. The precision plots for the KEGG and Reactome databases are presented. These plots illustrate the precision values obtained for different methods in relation to the benchmark datasets. The precision values reflect the accuracy of the methods in identifying interactions within the KEGG and Reactome pathways. It is worth noting that the precision curves provided are not precision–recall curves due to challenges in calculating recall based on pathways. The imbalance in the datasets is acknowledged as a concern for ROC curves, which is why the true positives and false positives are explicitly shown in the plots instead of normalizing the data.

S3. FAVA performs better on simulated datasets for Gene Regulatory Network predictions

Another common way to assess the performance of computational methods is using simulated data. A few simulators exist that can create simulated expression matrices from Gene Regulatory Networks (GRNs). Even though GRNs remain outside of the scope of

methods constructing functional association networks, we decided to evaluate their performance on simulated data created with two algorithms, namely scMultiSim (53) SERGIO (54). The results show that FAVA outperforms hdWGCNA, scLink, and PCC for prediction of GRNs from both simulation algorithms (**Figure S3)**. In both cases, FAVA has the best performance with a TP/FP ratio of 0.011 (116/9,884) and 0.02 (208/9,792) for the datasets simulated by scMultisim and SERGIO, respectively. For the scMultisim dataset, scLink gave the second best performance (74/9,926=0.007) (**Figure S3a)**, whereas the ranking of the other methods is less clear on the SERGIO dataset (**Figure S3b)**.

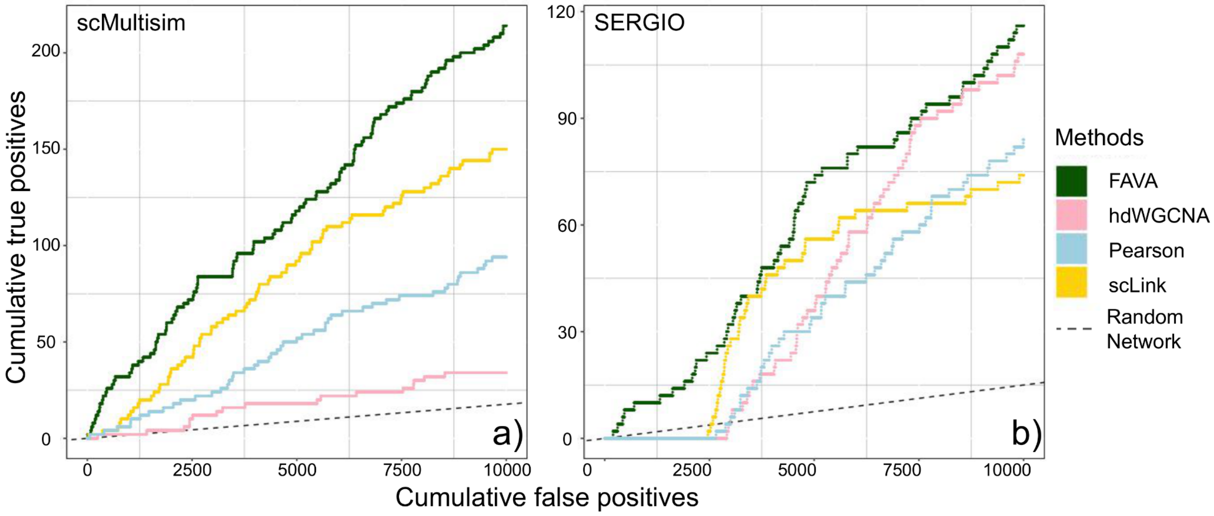

Figure S3. Performance of the methods on simulated data. a) Method performance on simulated data from the scMultisim package. FAVA outperforms the other algorithms with ~1.6 times better performance than the second best (scLink). b) Methods performance on simulated data from the SERGIO package. FAVA outperforms the other algorithms, whereas the ranking of the other methods is complicated.

We tried to also use a third simulation algorithm, BEELINE (55), which is a well-known framework for benchmarking the performance of computational methods in constructing GRNs. BEELINE contains pre-implemented algorithms dedicated to GRN prediction and suitable expression datasets and benchmarks. Unfortunately, we were not able to make BEELINE run correctly.

In our study, we recognize the fundamental distinction between GRNs and functional association networks, with the former focusing on regulatory interactions among genes and the latter encompassing a broader range of functional associations. Consequently, there is no fair way to compare methods designed to produce these two different types of networks. While benchmarking all available GRN methods on functional association benchmarks is possible, GRN methods should be expected to underperform compared to methods like FAVA, which produce much more inclusive functional association networks. Conversely, GRN methods ought to outperform FAVA on benchmark sets specifically tailored for regulon analysis.

S4. Influence of Cell Numbers on Network Quality in Tissues

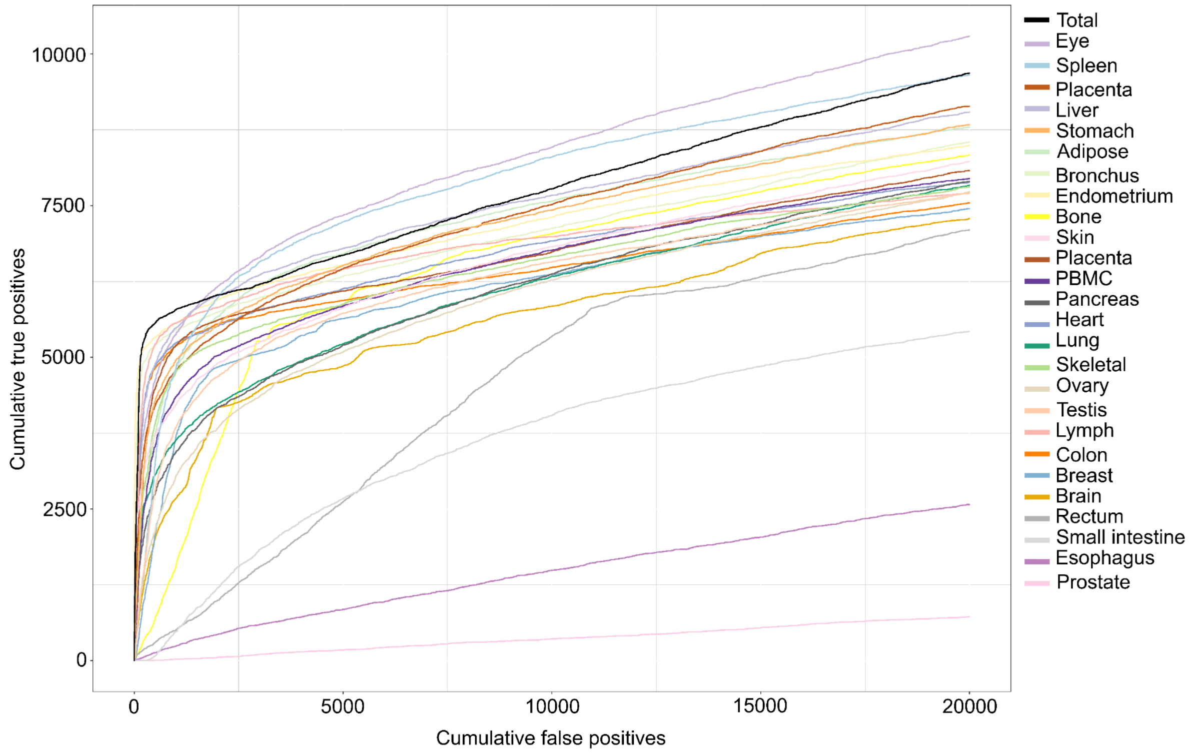

Tissue	No. of cells	Tissue	No. of cells
Adipose Tissue	83,535	Lymph	7,771
Bone	4,512	Ovary	43,636
Brain	76,533	Pancreas	3,719
Breast	47,662	PBMC (Blood)	4,972
Bronchus	17,521	Placenta	18,547
Colon	11,167	Prostate	35,861
Endometrium	71,032	Rectum	3,898
Esophagus	9,117	Skeletal	22,030
Eye	20,091	Skin	15,798
Heart	9,182	Small intestine	6,167
Kidney	25,279	Spleen	3,230
Liver	8,439	Stomach	5,318
Lung	4,599	Testis	6,490

Figure S4. Performance of FAVA Networks on KEGG Pathways. In our benchmarking analysis, we evaluated the performance of FAVA networks on KEGG pathways and observed that the majority of tissues yielded

high-quality networks. Surprisingly, we found that the quality of the networks did not straightforwardly correlate with the number of cells used. For instance, the spleen network demonstrated excellent performance despite being constructed from just 3,230 cells. This observation suggests that while having a larger number of cells can be advantageous, it does not necessarily guarantee superior network quality. The fact that the spleen network, with its limited cell count, produced one of the better networks underscores the complexity of the relationship between the number of cells and network performance in FAVA. These findings highlight the need for a nuanced understanding of the factors influencing network quality and support the notion that FAVA can generate robust networks even with modest cell numbers.

S5. Generalization of FAVA algorithm on unseen data

In FAVA, distinct VAEs are trained on separate datasets without overfitting, as evidenced by consistent performance on both training and test sets of a scRNA-seq dataset (GSE75748 (56)), with accuracy assessed using KEGG.

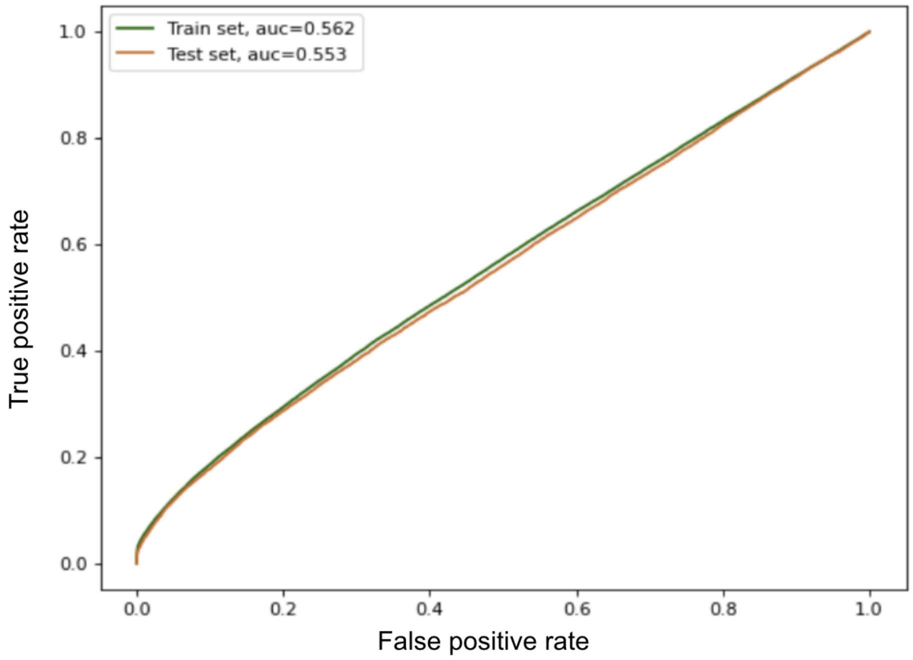

Figure S5. Generalization on unseen data. We split the scRNA-seq dataset (GSE75748) into a training (67%) and a test (33%) set. After training FAVA, the algorithm was applied on both the training and the test set to predict associations. The performance of the predictions is evaluated on KEGG (Materials and Methods). The plot shows that our framework is able to generalize when applied on unseen data (i.e. the test set). This is evident from the similarity between the green line (train set) and brown line (test set) in terms of true positive predictions. It is clear that even in the unseen 33% of the dataset, the VAE is able to capture patterns and that the latent representation can be used to predict existing interactions.

S6. Comparison of Spearman and Pearson correlation on bulk proteomics data

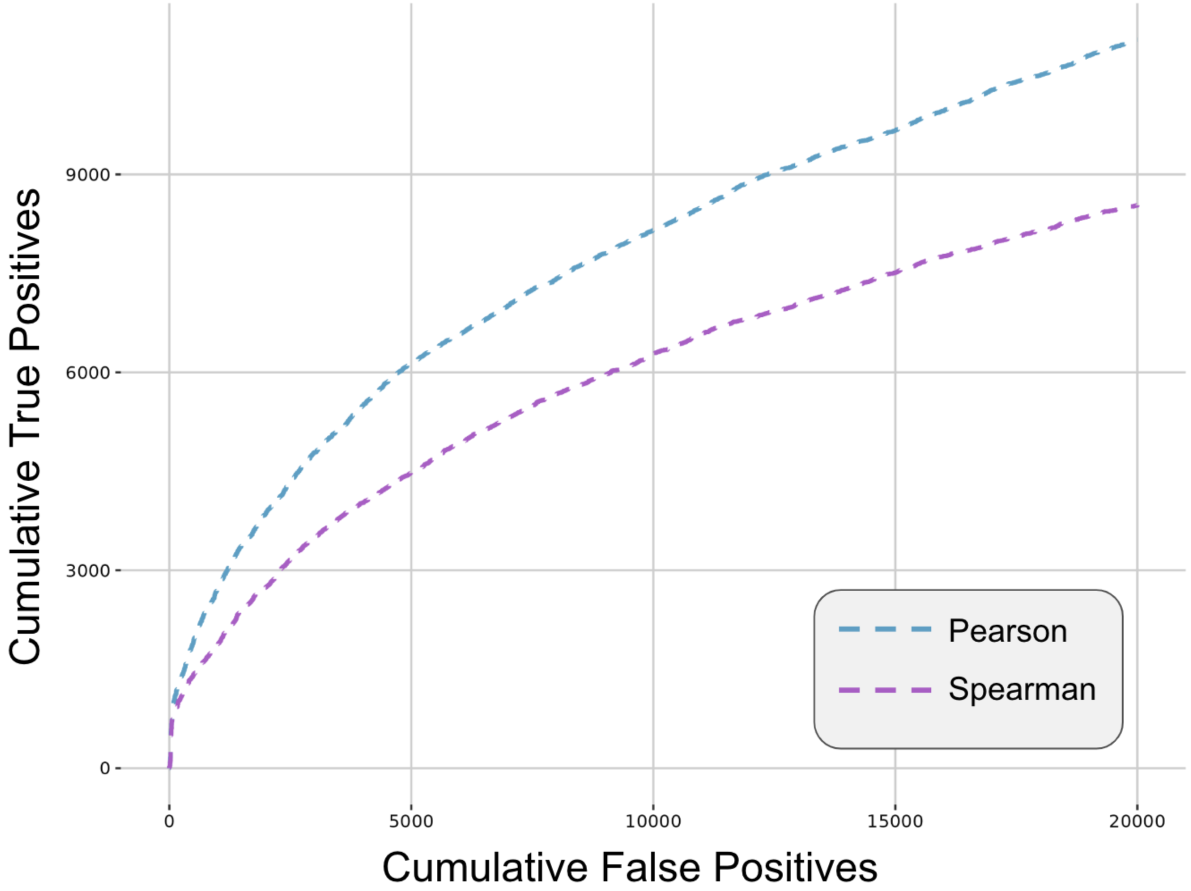

Figure S6. Spearman vs Pearson correlation on bulk proteomics data. This figure contrasts the prediction accuracy of Pearson and Spearman correlation methods using the PRIDE proteomics dataset, benchmarked against the KEGG database. The dashed lines represent the cumulative true versus false positives, with Pearson (blue) demonstrating slightly better performance, thus underscoring its selection as the baseline method for our analyses. It should be noted that Spearman's rank correlation is less sensitive to the presence of zeros and can better handle non-linear relationships. The similarity in outcomes from both Pearson and Spearman correlation analyses reinforces our confidence in the findings and suggests that the high percentage of zeros in our dataset does not significantly skew the results.

S7. Comparison of FAVA's combined network to hu.MAP 2.0.

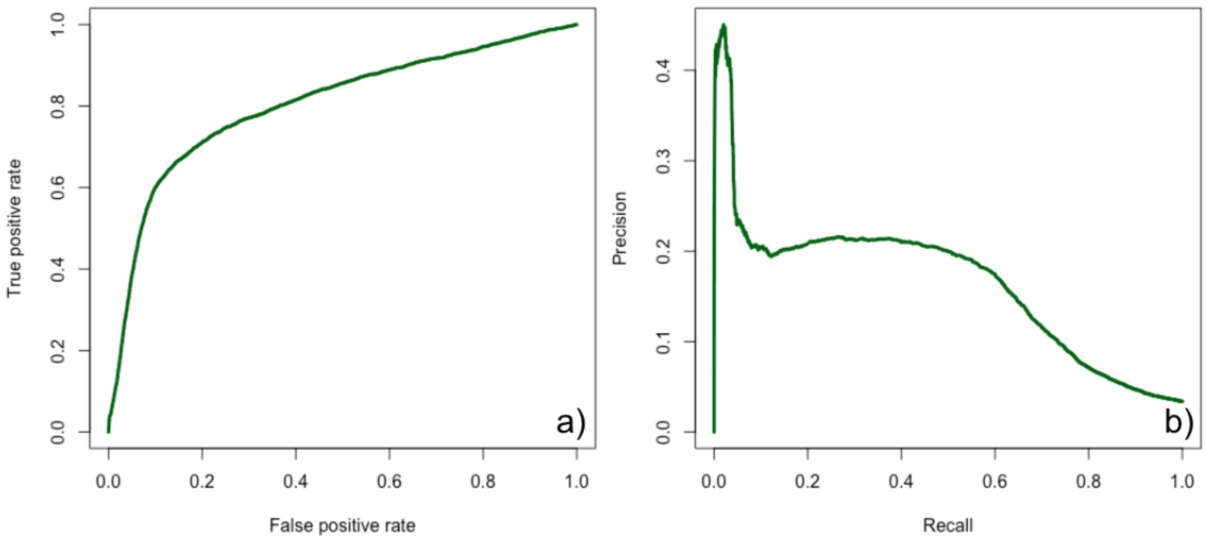

Figure S7. ROC curve, Precision and recall of FAVA's combined network based on hu.MAP 2.0. We conducted a comparison between our network from single-cells and bulk proteomics data and the hu.MAP 2.0 physical interaction network. We report the a) roc curve and the b) precision and recall calculations of our network on hu.MAP 2.0 edges, showing the agreement between the two networks.

S8. The Network

A *.pdf* containing the network from **Figure 4** with readable labels is provided.

S9. Knowledge gain from FAVA's combined network

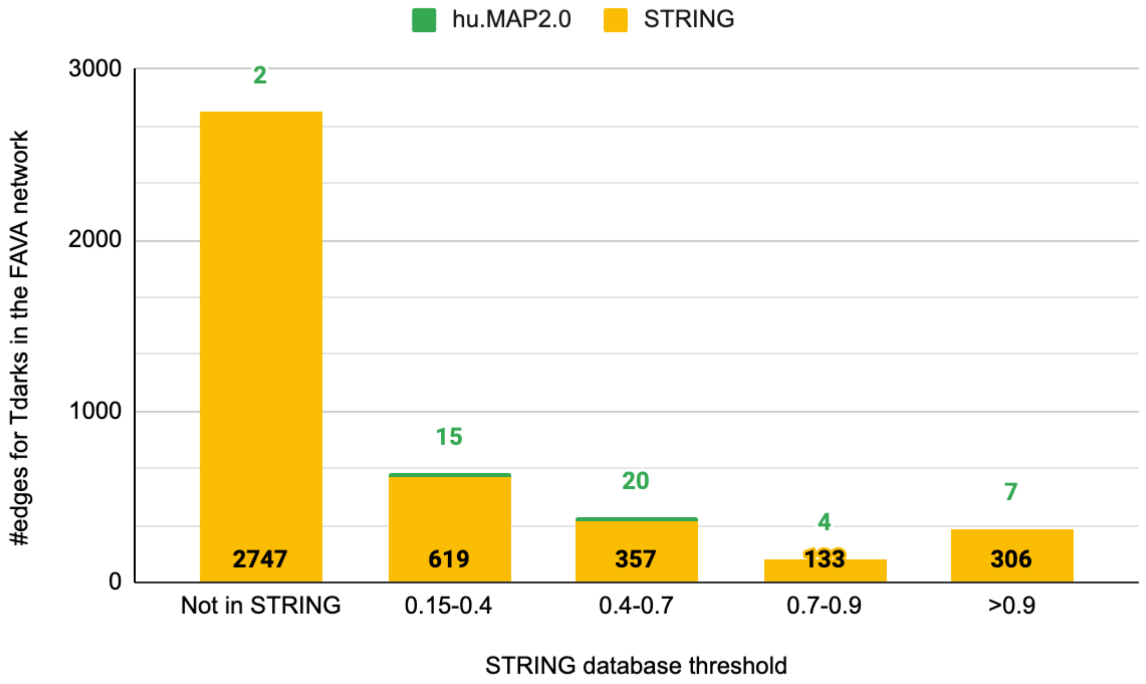

Figure S9. Characterization of the knowledge gain regarding understudied proteins. To assess what is the added value in terms of newly detected interactions by FAVA, we have compared the number of FAVA edges found in the STRING v11.5 databases and in hu.MAP 2.0. Four different score cutoffs were used for STRING (0.15 - includes all STRING edges, 0.4 - STRING medium confidence, 0.7 - STRING high confidence, 0.9 -

STRING very high confidence). The STRING network was used for this comparison because it is one of the most comprehensive networks according to a comparison of 21 large-scale molecular interaction networks (10.1016/j.cels.2018.03.001). The majority of the FAVA interactions for Tdark proteins cannot be found in STRING at any score cutoff, and only very few interactions are in hu.MAP 2.0. This showcases both the added value from using functional association data, but also the power of FAVA to detect these and further denotes the complementary nature of this network to current available experimental and literature knowledge. In addition we showcase complementarity to known resources. Specifically, 35% of interactions in the FAVA network exist in either huMAP2.0 or STRINGv11.5. This significant overlap serves as a strong indicator that our predictions are not merely generating false positives but are indeed uncovering biologically relevant interactions.

S10. The Cytoscape session of the network and the analysis.

Supplementary Table 1

The table presents the results of a per-cluster enrichment analysis conducted on the FAVA network. This Table contains the full results of the enrichment analysis for each cluster (**Sheet 1 - Full functional enrichment results**). Additionally, a condensed version of the table is provided (**Sheet 2 - Selected terms of each cluster**), highlighting the most significant enriched terms in seven distinct categories: Pathways, UniProt Keywords, Cellular Compartments, Tissues, Biological Processes, Molecular Functions, and Diseases. These tables offer valuable insights into the functional characteristics of each cluster in the FAVA network.

Supplementary Table 1 - FAVA
